# CFTR limits F‐actin formation and promotes morphological alignment with flow in human lung microvascular endothelial cells

**DOI:** 10.14814/phy2.15128

**Published:** 2021-12-01

**Authors:** Adam J. Causer, Maha Khalaf, Emily Klein Rot, Kimberly Brand, James Smith, Stephen J. Bailey, Michael H. Cummings, Anthony I. Shepherd, Zoe L. Saynor, Janis K. Shute

**Affiliations:** ^1^ Department for Health University of Bath Bath UK; ^2^ School of Pharmacy and Biomedical Sciences University of Portsmouth Portsmouth UK; ^3^ School of Sport, Health and Exercise Science University of Portsmouth Portsmouth UK; ^4^ School of Life Science, Engineering & Design Saxion University Enschede The Netherlands; ^5^ School of Sport, Exercise and Health Sciences Loughborough University Loughborough UK; ^6^ Department of Diabetes and Endocrinology Queen Alexandra Hospital Portsmouth UK

**Keywords:** actin cytoskeleton, cystic fibrosis, pulmonary microvascular endothelium, shear stress

## Abstract

Micro‐ and macrovascular endothelial dysfunction in response to shear stress has been observed in cystic fibrosis (CF), and has been associated with inflammation and oxidative stress. We tested the hypothesis that the cystic fibrosis transmembrane conductance regulator (CFTR) regulates endothelial actin cytoskeleton dynamics and cellular alignment in response to flow. Human lung microvascular endothelial cells (HLMVEC) were cultured with either the CFTR inhibitor GlyH‐101 (20 µM) or CFTRinh‐172 (20 µM), tumor necrosis factor (TNF)‐α (10 ng/ml) or a vehicle control (0.1% dimethyl sulfoxide) during 24 and 48 h of exposure to shear stress (11.1 dynes/cm^2^) or under static control conditions. Cellular morphology and filamentous actin (F‐actin) were assessed using immunocytochemistry. [Nitrite] and endothelin‐1 ([ET‐1]) were determined in cell culture supernatant by ozone‐based chemiluminescence and ELISA, respectively. Treatment of HLMVECs with both CFTR inhibitors prevented alignment of HLMVEC in the direction of flow after 24 and 48 h of shear stress, compared to vehicle control (both *p* < 0.05). Treatment with TNF‐α significantly increased total F‐actin after 24 h versus control (*p* < 0.05), an effect that was independent of shear stress. GlyH‐101 significantly increased F‐actin after 24 h of shear stress versus control (*p* < 0.05), with a significant (*p* < 0.05) reduction in cortical F‐actin under both static and flow conditions. Shear stress decreased [ET‐1] after 24 h (*p* < 0.05) and increased [nitrite] after 48 h (*p* < 0.05), but neither [nitrite] nor [ET‐1] was affected by GlyH‐101 (*p* > 0.05). CFTR appears to limit cytosolic actin polymerization, while maintaining a cortical rim actin distribution that is important for maintaining barrier integrity and promoting alignment with flow, without effects on endothelial nitrite or ET‐1 production.

## Graphical abstract

Cystic fibrosis transmembrane conductance regulator (CFTR) in human lung microvascular endothelial cells (HLMVEC) appears to limit cytosolic actin polymerization while maintaining a cortical rim filamentous actin (F‐actin) distribution, as demonstrated by staining F‐actin with rhodamine phalloidin (red) under static conditions in the absence and presence of the CFTR inhibitor GlyH‐101. The CFTR inhibitor induced loss of the cortical rim F‐actin leading to loss of cell‐cell contacts and reduced alignment with flow, without effects on endothelial nitrite or endothelin‐1 production.

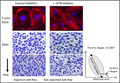

## 1 INTRODUCTION

Cystic fibrosis (CF) is a multi‐organ, life‐shortening autosomal recessive disease that affects 90–100,000 people worldwide (Bell et al., 2020). The etiology of CF is a mutation of the gene encoding the CF transmembrane conductance regulator (CFTR) protein, which was first described in 1989 by Kerem et al. (1989), Riordan et al. (1989), and Rommens et al. (1989) and is commonly expressed within epithelial cells where it normally functions as an anion, predominantly chloride ion, channel (Elborn, 2016). However, expression of CFTR has also been reported in human lung microvascular endothelial cells (HLMVEC; Khalaf et al., 2020; Tousson et al., 1998), human umbilical vein endothelial cells (HUVEC; Declercq et al., 2021; Tousson et al., 1998) and human pulmonary artery endothelial cells (HPAEC; Plebani et al., 2017; Totani et al., 2017). Furthermore, microvascular and macrovascular endothelial perturbations are evident in people with CF (Poore et al., 2013; Rodriguez‐Miguelez et al., 2016) and progress with the age of the patient (Kreslová et al., 2021).

The endothelium forms a semi‐permeable barrier between tissues and the blood stream, and is essential for regulating vascular tension, molecular transport, and immune cell trafficking, functions that differ depending on the vessel and location of the vascular bed (reviewed by Krüger‐Genge et al., 2019), and have been described in HLMVECs (Blease et al., 1998; Catravas et al., 2010; Sedgwick et al., 2002; Yamamoto et al., 1998). In vivo endothelial cells are constantly exposed to shear stress resulting in the activation of several mechanoresponsive networks, which increase the expression of genes that regulate inflammation, such as the vascular cell adhesion molecule (Zhou et al., 2014) and bioavailability of the vasodilator nitric oxide (NO), such as endothelial NO synthase (eNOS) (Chistiakov et al., 2017). In CF, defective flow‐mediated dilation (FMD) of the brachial artery suggests NO bioavailability is reduced in response to shear stress (Poore et al., 2013), and improved by agents that increase NO availability (Rodriguez‐Miguelez et al., 2018). This notion is further supported by the observation that acute supplementation with 20 mg/kg of oral tetrahydrobiopterin (an essential cofactor for eNOS activity) significantly improved FMD, through reduced superoxide production and increased NO, which is indicative of improved eNOS coupling in people with CF (Jeong et al., 2018).

The endothelial actin cytoskeleton, which comprises a cortical rim, cytosolic stress fibers and the membrane skeleton (Prasain & Stevens, 2009) is remodeled on exposure to shear stress. Filamentous actin (F‐actin) redistributes from a dense band at the periphery of the cell to create bundles of intracellular stress fibers, and both cells and stress fibers align with the direction of flow (Dardik et al., 2005; Davies, 1995). Cortical rim F‐actin is essential for facilitating cell‐to‐cell interaction and function, by tethering cell‐matrix complexes such as vascular endothelial (VE)‐cadherin (Efimova & Svitkina, 2018) and eNOS (Su et al., 2005). Inflammatory mediators (Ehringer et al., 1999), including tumor necrosis factor (TNF)α (Wójciak‐Stothard et al., 1998) and oxidative stress (Huot et al., 1997), cause the formation of F‐actin cytosolic stress fibers thereby increasing cellular stiffness and limiting NO release (Fels et al., 2012).

Impaired function of CFTR in HLMVECs (Khalaf et al., 2020) and in HUVECs (Declercq et al., 2021) in vitro induces a pro‐inflammatory phenotype and increased oxidative stress. Functional CFTR is reported to limit inflammation and oxidative stress by upregulating the expression of nuclear factor erythroid 2‐related factor 2 (Nrf2) in the epithelium (Borcherding et al., 2019; Chen et al., 2008) and endothelium (Khalaf et al., 2020). It is, therefore, hypothesized that a loss of endothelial CFTR function may contribute to a pro‐inflammatory and pro‐oxidant state that causes a re‐organization of the F‐actin cytoskeleton and, subsequently, reduce NO bioavailability.

The purpose of the present study was to investigate the effect of pharmacological CFTR inhibition on actin cytoskeletal distribution and morphological alignment of cells in response to shear stress in vitro, and on the release of the vasodilator NO, measured as the physiological metabolite nitrite, and the potent vasoconstrictor endothelin (ET)‐1 from HLMVEC under static and flow conditions.

## 2 MATERIALS AND METHODS

All reagents were purchased from Merck & Co. (NJ, USA) unless indicated otherwise.

### 2.1 Culture of HLMVEC

Human lung microvascular endothelial cells (Lonza Biologics) were maintained in 5% CO_2_ at 37°C and used until passage 9. Cells were grown in Lonza EGM‐2 MV full growth medium (FGM; CC‐3202), which consisted of basal medium (CC‐3156) and supplemental EGM‐2 MV (CC‐4147). The supplemental media consisted of (final concentration) 5% (v/v) fetal bovine serum, 0.4% (v/v) hydrocortisone, 4% (v/v) human fibroblast growth factor, 1% (v/v) vascular endothelial growth factor (VEGF), 1% (v/v) R3‐IGF‐1, 1% (v/v) ascorbic acid, 1% (v/v) human epidermal growth factor, and 1% (v/v) gentamycin and amphotericin B (GA‐1000).

Human lung microvascular endothelial cells (250 × 10^3^ cells/well in 1 ml of FGM) were seeded in six‐well plates coated with human collagen IV (0.1 mg/ml). A 15 mm diameter adhesive silicon disk was attached to the center of each well to prevent cell attachment in the center of the well, while allowing cells to adhere and grow in the periphery. After 24 h, this disk was removed and cells were then treated with 2 ml of FGM containing either CFTRinh‐172 (20 μM), a concentration that was previously shown to significantly inhibit the barrier function of human and sheep arterial endothelial cell layers (Brown et al., 2014) or GlyH‐101 (20 μM), the concentration we have previously shown to specifically inhibit CFTR activity, induce oxidative stress and pro‐inflammatory cytokine production (Khalaf et al., 2020), TNF‐α (10 ng/ml, Peprotech) which is reported to increase filamentous F‐actin and remodel EC morphology as a positive control (Marcos‐Ramiro et al., 2014) at a concentration we previously showed, induces oxidative stress and pro‐inflammatory cytokine production in HLMVECs (Khalaf et al., 2020) or a negative vehicle control (0.1% [v/v] dimethyl sulfoxide; DMSO), with exposure to either 8, 16, 24 or 48 h of static incubation or orbital flow conditions at a speed of 210 rpm, which results in 11.1 dynes/cm^2^ of shear stress at the periphery of the well (Dardik et al., 2005).

### 2.2 Crystal violet staining

Human lung microvascular endothelial cells were washed with 2 ml/well of phosphate buffered saline (PBS; −Ca/−Mg), and subsequently fixed with 2 ml/well of fresh 4% (w/v) paraformaldehyde (in PBS [–Ca/–Mg]) for 2 min. The 4% (w/v) paraformaldehyde was then removed, and cells were washed twice with 2 ml/well of PBS (–Ca/–Mg). Cells were then stained with 1 ml/well of 0.125% (w/v) crystal violet (in ultra‐high quality water) for 10 min. The 0.125% (w/v) crystal violet was then removed, and cells were washed three times with 2 ml/well of PBS (–Ca/–Mg). Data were collected from single wells of three independent experiments for the 24 and 48 h time points. Flow alignment was not observed at the earlier, 8 and 16 h, time points.

**FIGURE 1:**
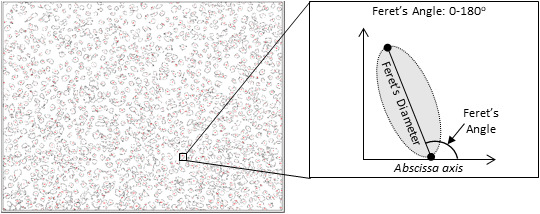
The calculation of Feret's Angle of human lung microvascular endothelial cells. After the nucleus was stained with 0.125% crystal violet, the direction of alignment was quantified by identifying the angle formed by the longest axis of the cell (Feret's Diameter) with the abscissa axis (Feret's Angle). Each image contained >500 cells and images were collected from two randomly selected areas in each well under each incubation condition in three independent experiments

Human lung microvascular endothelial cells were imaged in duplicate (at a magnification of 10×) at the periphery of the well, where flow was horizontal or vertical. ImageJ software (National Institute for Health) was used to quantify changes in cellular morphology. Specifically, RGB images were converted to gray scale (8‐bit) and made binary. Following this, cells were identified as being ≥200 pixels in size. The direction of alignment was quantified by identifying the angle formed by the longest axis of the cell (Feret's Diameter) with the abscissa axis (Feret's Angle; Figure 1; Fioretta, 2014; Thodeti et al., 2009). The Feret's Angle ranges from 0° to 180° and a reduced variance of the Feret's Angle within an image of >500 cells was indicative of a greater alignment with flow.

Crystal violet was subsequently extracted from the cells to estimate the number of adherent viable cells remaining after treatment with GlyH‐101, TNF or control, under static or shear stress conditions. Specifically, 2 ml ethanol/acetone (80/20%) was added to each well and incubated for 5 min at room temperature. Following this, 200 μl was transferred from each well into a 96‐well plate and absorbance was measured at 550 nm.

### 2.3 F‐actin staining

Human lung microvascular endothelial cells were washed with 2 ml/well of PBS (–Ca/–Mg), and subsequently fixed with 2 ml/well of fresh 4% paraformaldehyde (in PBS [–Ca/–Mg]) for 10 min. The 4% (w/v) paraformaldehyde was then removed, and cells were washed twice with 2 ml/well of PBS (–Ca/–Mg). Cells were then permeabilized with 2 ml/well of 0.1% (v/v) Triton X‐100 (in PBS [–Ca/–Mg]) for 5 min. The 0.1% (v/v) Triton X‐100 was then removed and cells were washed twice with 2 ml/well of PBS (–Ca/–Mg). Cells were then incubated at room temperature in 660 μl/well of 100 nM of rhodamine phalloidin (ThermoFisher; prepared in PBS [–Ca/–Mg] containing 1% [w/v] bovine serum albumin) for 20 min, followed by an additional washing step. Subsequently, cells were co‐stained with 1 ml/well of 5 μg/ml Hoechst 33342 (Molecular Probes) for 10 min. For the initial dose‐ and time‐dependent analysis of F‐actin responses to GlyH‐101 under static conditions, data were collected from duplicate wells of three independent experiments at 16, 24, and 48 h. To investigate the effect of shear stress on F‐actin responses versus static conditions, data from three peripheral areas of a single well from three independent experiments at the 24 h time point were used for the quantitative and qualitative analysis of the effect of shear stress on F‐actin. After 48 h incubation the cells appeared intolerant to the F‐actin staining procedure and (except in one experiment) for reasons that are not known, but may reflect the permeabilization step on cells less adherent at this time point, were largely dislodged from the plate. F‐actin data are therefore shown only for the 24 h time point.

Imaging was performed using a Zeiss Axiovert 200M (inverted) microscope (Carl Zeiss) contained in an incubator (37°C, 5% CO_2_, humid atmosphere). Objectives (air) 10× and 20× were used to obtain bright field and fluorescent images (Volocity 5.4, Quorum Technologies). For the latter, the following filter sets were used: exc. 365 nm, em. 445/50 nm for Hoechst 33342 staining of nuclei, and exc. 546/12 nm, em. 575–640 nm for rhodamine staining of F‐actin, with 400 ms fluorescence excitation used to capture the images in Figure 4, and 200 ms exposure for the semi‐quantitative analysis of F‐actin in all cases. In some cases, images were obtained and over‐layed using Volocity software (V6.1.1, Ontario, Canada). ImageJ was also used to quantify Rhodamine Phalloidin and Hoechst 33342 staining. Specifically, RGB color images were converted to a RGB stack; with red fluorescence measured between 20 and 255 thresholds and blue fluorescence measured between 40 and 255 thresholds. RawIntDen of red was expressed relative to RawIntDen of blue for the semi‐quantitative analysis of F‐actin per cell.

ImageJ was also used to assess changes in the cellular distribution of F‐actin between peripheral (cortical) and cytoplasmic F‐actin. The ‘Edges’ tool was used to highlight peripheral F‐actin staining in the red channel, and the ‘Plot Profile’ data for three straight lines drawn side‐to‐side across each image was obtained, which analyzed the staining intensity in pixels across the profile and plots the profile as a line graph. The *x*,*y* data were copied into GraphPad Prism to calculate the area under the curve for the profile peaks (the peripheral staining), after background subtraction for the intensity of staining in the cytoplasm.

### 2.4 Measurement of [nitrite] and [ET‐1]

Supernatant (2 ml) was harvested from duplicate wells of three independent experiments and centrifuged at 1500× g for 10 min at 4°C. Acellular supernatant was then frozen and stored at −80°C for future analysis.

Supernatant [nitrite] was measured using Sievers NOA 280i (Analytix Ltd.), which is a chemiluminescence‐based NO analyzer. Specifically, 150 μl of undiluted sample was injected into a purge vessel containing 5 ml of 4% (w/v) glacial acetic acid and sodium iodide solution which reduced nitrite to NO gas. The resultant NO gas was carried into the reaction cells of the analyzer with inert nitrogen gas wherein NO gas reacted with ozone to form nitrogen dioxide in the excited state. Upon return to its ground state, electronically excited nitrogen dioxide emitted a photon that was detected by red‐sensitive photomultiplier tube. The concentration of nitrite in the samples was calculated from a standard plot of known sodium nitrite concentrations.

Supernatant [ET‐1] was measured by a commercially available ET Pan Specific ELISA Kit (DY1160; Abcam) with ET‐1 standards. Samples were diluted 1:2 before loading to the plates and data were transformed to concentration, according to a standard curve ranging from 3.91 to 250 pg/ml. The ELISA was performed as per the manufacturer's instructions.

### 2.5 Statistical analysis

Data were presented as mean ± SEM of *n* independent experiments, with replicate data averaged in each independent experiment, unless otherwise stated. Statistical analysis was carried out using GraphPad Prism version 8 software. Two‐way ANOVA was used to determine exposure to the effects of (static or orbital shaking) x condition (vehicle control, GlyH‐101, CFTRinh‐172 or TNF‐α) for cell viability, cellular alignment, and actin polymerization. Differences in peripheral F‐actin staining were analyzed by two‐way ANOVA. Two‐way ANOVA was also used to determine the effect of time (8, 24, and 48 h) × exposure (static or orbital shaking) on supernatant [nitrite] and [ET‐1] to determine the efficacy of the shear stress model. A three‐way ANOVA was used to determine the interaction effect of time (24 and 48 h) × exposure (static or orbital shaking) × condition (vehicle control, GlyH‐101, and TNF‐α) on supernatant [nitrite] and [ET‐1]. Fishers LSD post‐hoc *t*‐tests were also used to determine the effect of GlyH‐101 upon outcomes during both static and orbital incubation. Effect sizes were estimated using Cohen's *d*.

## 3 RESULTS

### 3.1 Cell viability

Exposure to 24 h of either static or shear incubation did not significantly change estimated cell viability in the absence or presence of CFTR inhibitor (all *p* > 0.05). However, extraction of crystal violet demonstrated that the estimated cell count was significantly reduced after 48 h treatment with GlyH‐101 compared to DMSO (*n* = 3, *p* < 0.01) and TNFα (*n* = 3, *p* < 0.01), and the effect was the same magnitude under both static and orbital conditions (Figure S1).

### 3.2 Alignment with flow

**FIGURE 2:**
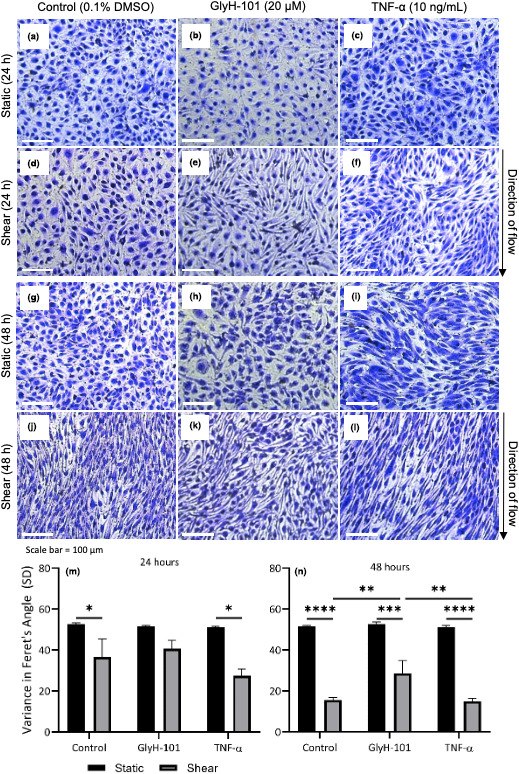
Human lung microvascular endothelial cells alignment in response to static (black bars) or shear stress (11.1 dynes/cm^2^; gray bars) incubation, and following treatment with vehicle control (0.1% dimethyl sulfoxide [DMSO]), GlyH‐101 (20 μM), or TNF‐α (10 ng/ml) for 24 or 48 h. Nuclei were stained with 0.125% crystal violet and images were captured at 10× magnification. Uniformity of alignment was quantified by determining the SD of the Feret's Angle for each cell within an image (>500 cells per image). Images were captured from two separate areas in a single well of three independent experiments (*n* = 3). HLMVECs were incubated under static conditions for 24 h (a, b, c) or 48 h (g, h, i), or under orbital flow conditions for 24h (d, e, f) or 48 h (j, k, l), in the absence (a, d, g, j) or presence of GlyH‐101 (b, e, h, k) or TNF‐α (c, f, i, l). Analysis of the variance of Feret's angle at 24h (m) and 48h (n) is shown for each of these conditions. Data were expressed as mean ± SEM. *p < 0.05; **p < 0.01; ***p < 0.001; ****p < 0.0001

Representative images of HLMVEC morphology following static or orbital incubation, and in response to GlyH‐101, TNF‐α, or vehicle control treatments are presented in Figure 2. Following 24 h of shear stress, cells treated with vehicle control or TNF‐α (both *p* < 0.05), but not GlyH‐101 (*p* > 0.05), were significantly aligned with flow as the variance in the Feret's Angle was significantly reduced compared to static conditions (Figure 2m). After 48 h of shear stress, cells treated with vehicle control, GlyH‐101, and TNF‐α all significantly aligned in the direction of flow compared to static conditions (Figure 2n, all *p* < 0.05). However, in cells treated with GlyH‐101 there was significantly greater variance in Feret's Angle, indicating reduced alignment with flow, compared with vehicle control and TNF‐α conditions at 48 h (Figure 2n, both *p* < 0.05).

**FIGURE 3:**
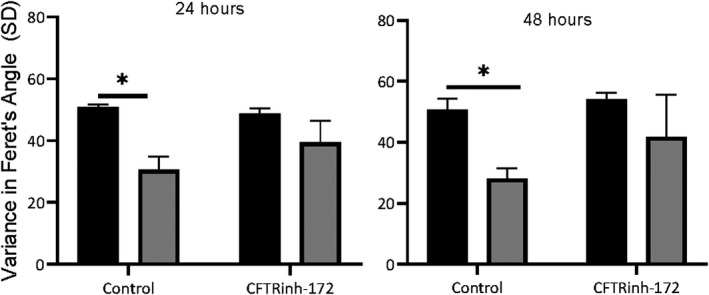
Human lung microvascular endothelial cells alignment in response to static (black bars) or shear stress (11.1 dynes/cm^2^; gray bars) incubation for 24 or 48 h, and following treatment with vehicle control (0.1% dimethyl sulfoxide) or CFTRinh‐172 (20 μM). Nuclei were stained with 0.125% crystal violet and images were captured at 10× magnification. Uniformity of alignment was quantified by determining the SD of the Feret's Angle for each cell within an image (>500 cells per image). Images were captured from two separate areas in a single well of three independent experiments (*n* = 3). Data were expressed as mean ± SEM. *p < 0.05

The effect of the CFTR inhibitor GlyH‐101 on HLMVEC alignment with flow, was confirmed using the CFTR inhibitor CFTRinh‐172, which also prevented the alignment of cells in the direction of flow (Figure 3).

### 3.3 F‐actin formation

In initial experiments under static conditions to investigate the dose‐response to GlyH‐101, there was a significant increase in F‐actin formation, measured as total F‐actin per cell, in cells treated with GlyH‐101 (20 μM) for 16 h (*n* = 3; 1.48 ± 0.29 RawIntDen F‐actin/nuclei, *p* < 0.05) versus vehicle control (*n* = 3; 0.89 ± 0.07 RawIntDen F‐actin/nuclei). However, there were no significant effect of treatment with 5 μM (*n* = 3; 0.97 ± 0.04 RawIntDen F‐actin/nuclei, *p* > 0.05 vs. control) or 10 μM (*n* = 3; 1.19 ± 0.14 RawIntDen F‐actin/nuclei, *p* > 0.05 vs. control) GlyH‐101 for 16 h versus vehicle control (Figure S2). Therefore, treatment with 20 μM GlyH‐101 was used in subsequent experiments.

**FIGURE 4:**
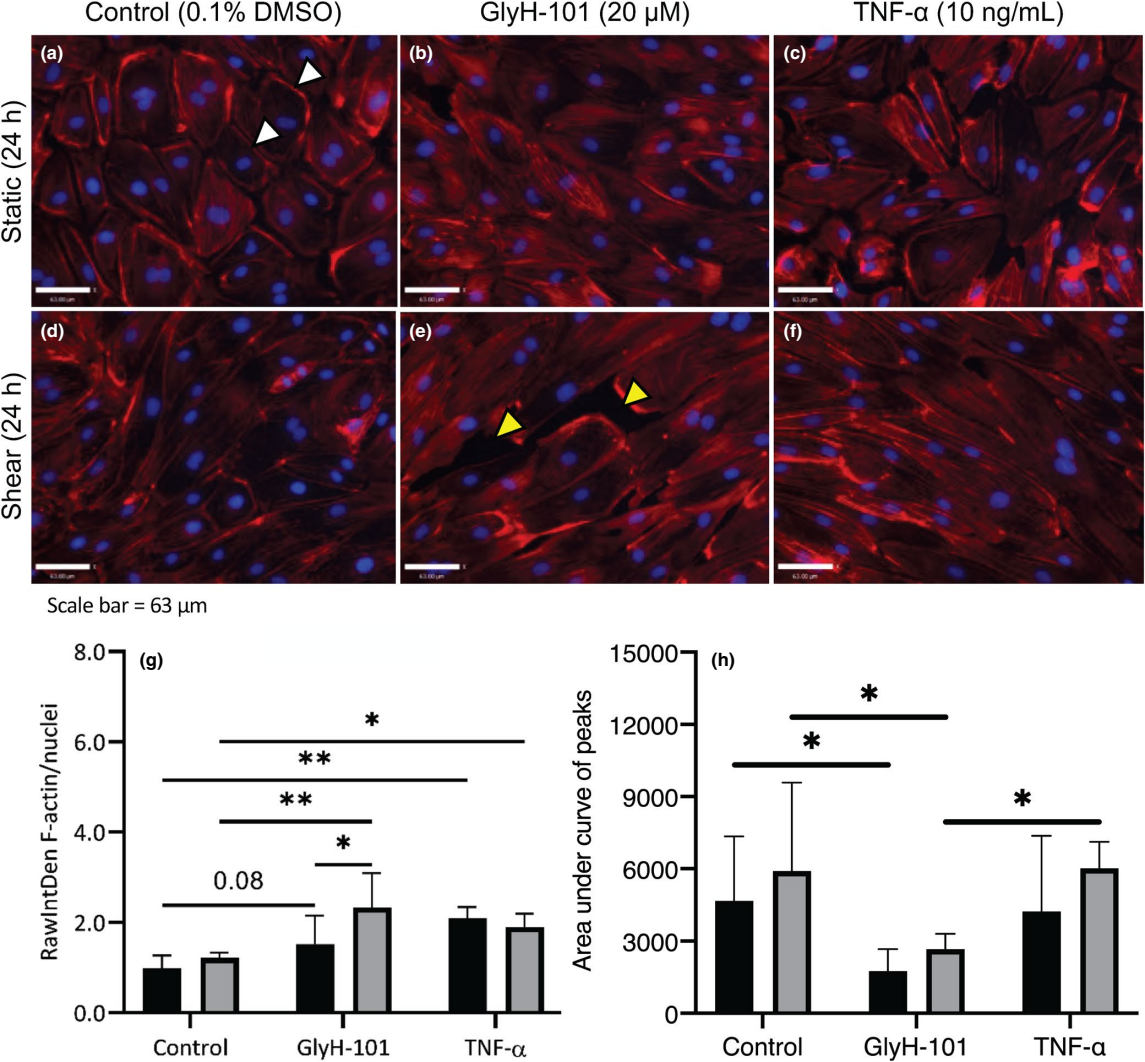
Human lung microvascular endothelial cells filamentous actin (F‐actin) formation in response to static (black bars) or shear stress (11.1 dynes/cm^2^; gray bars) incubation, and following treatment with vehicle control (0.1% dimethyl sulfoxide [DMSO]), GlyH‐101 (20 μM) or TNF‐α (10 ng/ml) for 24 h. Images included in this figure were captured at 20× magnification with 400 ms exposure to fluorescence (a–f). Images used for quantification were exposed to 200 ms of fluorescence. Images were captured from three randomly selected areas of a single well in three independent experiments (*n* = 3). The RawIntDen of F‐actin (Red; 100 nM of Rhodamine Phalloidin) was expressed relative to nuclei (blue; 5 μg/ml Hoechst 33342) to estimate F‐actin per cell (g). Peripheral F‐actin was analyzed using the ‘Plot Profile’ tool in ImageJ and expressed as the area under the curve for the peaks in the profile representing peripheral F‐actin (h). Data were expressed as mean ± SEM. Yellow arrows highlight paracellular gaps; white arrows highlight cortical rim F‐actin; *p < 0.05; **p < 0.01

Representative images of F‐actin following static or orbital incubation for 24 h, and in response to GlyH‐101, TNF‐α, or vehicle control treatments are presented in Figure 4. In cells treated with the vehicle control under static conditions, F‐actin was primarily distributed at the cortical rim (Figure 4a). Shear stress, alone (Figure 4d), did not increase total F‐actin in control cells, compared to static incubation (Figure 4a) at 24 h (*p* > 0.05; Figure 4g), and there was no effect of shear stress alone on the distribution of F‐actin between periphery and cytoplasm (Figure 4h). Under static conditions, TNF‐α treatment for 24 h (Figure 4c) induced a significant increase in total F‐actin compared to the vehicle control (Figure 4g, *p* < 0.05), with the cellular distribution remaining unchanged (Figure 4h). Despite the presence of a moderate effect size (Cohen's *d* = 0.64) there was no significant difference (*p* = 0.08) in total F‐actin content between cells treated with the vehicle control and GlyH‐101 under static conditions at 24 h. Under orbital conditions of shear stress, both GlyH‐101 and TNF‐⍺ increased F‐actin at 24 h compared to the vehicle control (Figure 4g, *p* < 0.05).

On treatment with GlyH‐101, there appeared to be a redistribution of F‐actin from the periphery (Figure 4b,e). Profile analysis of the images using ImageJ confirmed a loss of cortical rim F‐actin to form cytosolic F‐actin polymers in cells treated with GlyH‐101 at 24 h under both static and shear conditions (Figure 4h). Furthermore, a significant (*p* < 0.05) increase in F‐actin (Figure 4g) and the occurrence of paracellular gaps (Figure 4e) after 24 h of shear stress was observed in cells treated with GlyH‐101, suggesting the formation of cytosolic stress fibers and cellular retraction, compared to cells treated with GlyH‐101 in static conditions. However, this effect of shear stress was not seen in control or TNF‐α‐ stimulated conditions (Figure 4g).

### 3.4 Supernatant [nitrite] and [ET‐1]

**FIGURE 5:**
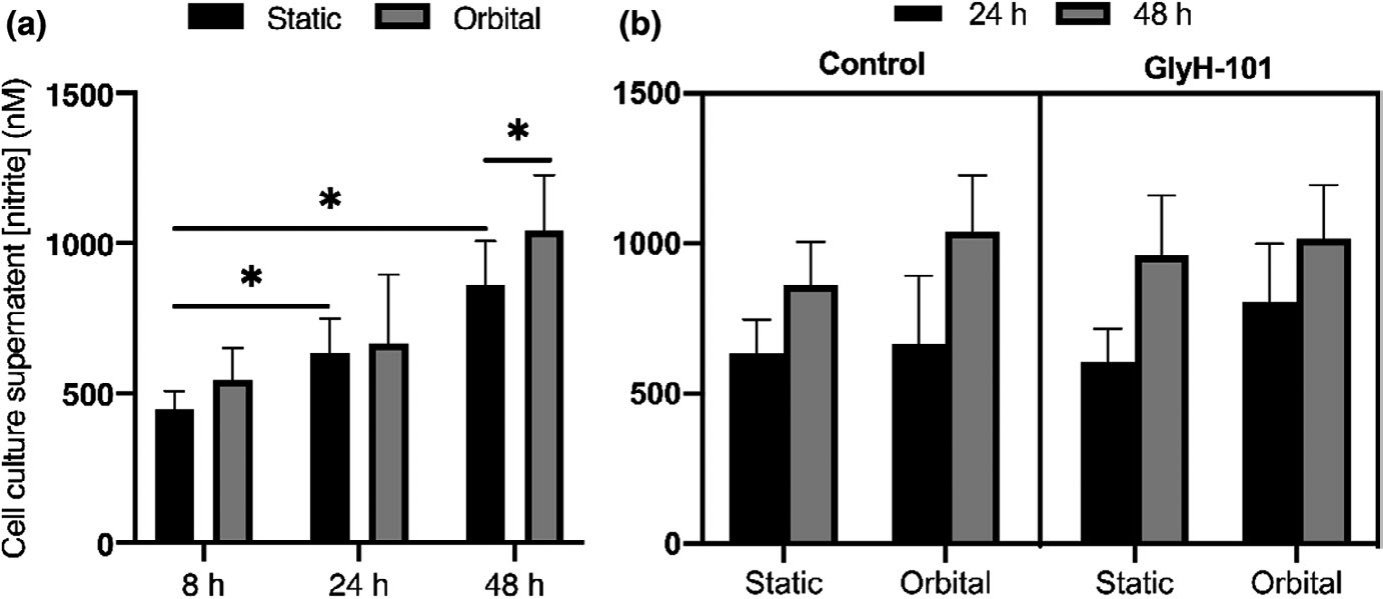
Human lung microvascular endothelial cells culture supernatant nitrite concentrations in response to static or shear stress (11.1 dynes/cm^2^) incubation over time (a), and following treatment with vehicle control (0.1% dimethyl sulfoxide) or GlyH‐101 (20 μM; b). Samples were collected from duplicate wells of three independent experiments (*n* = 3). Data were expressed as mean ± SEM. *p < 0.05; **p < 0.01

We further investigated the effect of CFTR inhibition on release of the potent vasodilator NO, measured as nitrite, and of the potent vasoconstrictor endothelin‐1 (ET‐1) in response to shear stress. Cell culture supernatant [nitrite] significantly increased with time over 24 and 48 h, under both static conditions versus 8 h (Figure 5a, *p* < 0.05). Shear stress only increased [nitrite] at 48 h (Figure 5a, *p* < 0.05). Post‐hoc analysis revealed that treatment of HLMVEC with GlyH‐101 had no significant effect on supernatant [nitrite] at any time point or cell culture condition (Figure 5b, all *p* > 0.05).

**FIGURE 6:**
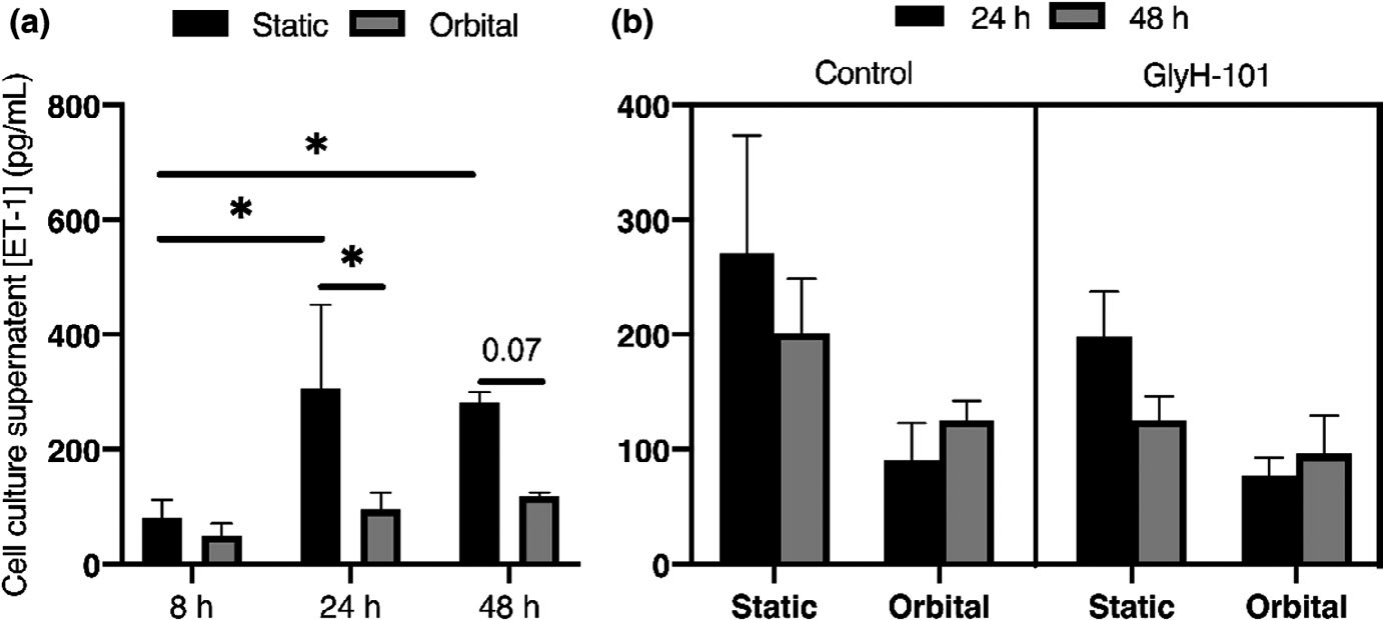
Human lung microvascular endothelial cells culture supernatant ET‐1 concentrations in response to static (black bar) or shear stress (gray bar; 11.1 dynes/cm^2^) incubation over time (a), and following treatment with vehicle control (0.1% dimethyl sulfoxide) or GlyH‐101 (20 μM) for 24 h (black bar) and 48 h (gray bar; b). Samples were collected from duplicate wells of three independent experiments (*n* = 3). Data were expressed as mean ± SEM

Cell culture supernatant [ET‐1] was significantly greater following 24 and 48 h incubation under static conditions versus 8 h static incubations (Figure 6a, both *p* < 0.05). Furthermore, shear stress significantly reduced [ET‐1] compared with static incubation for 24 h (*p* < 0.05), but not 48 h despite a large effect size being observed (Figure 6a; *p* = 0.07; Cohen's *d* = 6.88). Similar to [nitrite], post‐hoc analysis revealed that treatment of HLMVEC with GlyH‐101 had no significant effect on supernatant [ET‐1] at any time point or exposure (Figure 6b, all *p* > 0.05).

## 4 DISCUSSION

This is the first study to investigate the role of functional CFTR in the regulation of the actin cytoskeleton and cellular alignment of endothelial cells in response to flow. The present study identified three key findings: (1) pharmacological inhibition of endothelial CFTR significantly exacerbated actin polymerization in response to flow, through dismantling cortical rim F‐actin to form cytosolic stress fibers; (2) pharmacological inhibition of endothelial CFTR significantly disturbed cellular alignment in response to flow; and, (3) CFTR‐regulated adaptations to flow appeared to be independent of NO and ET‐1, as pharmacological inhibition of endothelial CFTR did not significantly alter cell culture supernatant [nitrite] or [ET‐1] during static or orbital incubation conditions. These findings are particularly important, given the growing body of evidence to support endothelial vascular perturbations in CF (Poore et al., 2013; Rodriguez‐Miguelez et al., 2016).

The present study employed a model of orbital flow, which was previously used to demonstrate that bovine aortic endothelial cells exposed to chronic shear stress undergo F‐actin redistribution resulting in modification in cellular morphology in the direction of flow (Dardik et al., 2005). The application of 11.1 dynes/cm^2^ shear stress reflects the relatively high shear stress seen in microvessels, compared to <1 dynes/cm^2^ measured in large veins (Ballermann et al., 1998). Exposure of normal endothelial cells to both 24 and 48 h of orbital shear stress in the present study promoted cellular alignment in the direction of flow (Figure 2m,n). These observations agree with those of Osborn et al. (2006) who reported that shear stress resulted in elongation and flow alignment of bovine aortic endothelial cells within 24 h.

The present study reported no statistically significant changes in total F‐actin content in normal endothelial cells that were exposed to shear stress alone. Further, a redistribution of F‐actin from the cortical rim to create bundles of stress fibers was not observed, as previously reported for normal human arterial and venous endothelial cells exposed to relatively high shear stress forces, but not the low shear forces seen in veins (0.5 dynes/cm^2^; Davies, 1995; Drenckhahn et al., 1986; Franke et al., 1984). The formation of endothelial stress fibers was proposed to protect endothelial cells from hydrodynamic damage or detachment at high shear stress in HUVEC (Drenckhahn et al., 1986).

Exposure to flow for 24–48 h resulted in a reduction of supernatant [ET‐1] in normal endothelial cells that was significant at 24 h (Figure 6a). Previous reports indicate that while acute shear stress, for 30 min to 6 h, can activate ET‐1 gene transcription in a process related to acute cytoskeletal remodeling and depolymerization of F‐actin to G‐actin (Morita et al., 1993; Yoshizumi et al., 1989), sustained shear stress over 6–24 h results in a profound reduction of ET‐1 gene expression and ET‐1 peptide release (Malek et al., 1993; Malek & Izumo, 1992; Morita et al., 1993), as observed in our experiments.

Conversely, exposure to shear stress resulted in a significantly higher supernatant [nitrite] in normal endothelial cells that were exposed to shear stress over 48 h (Figure 5a). Shear stress upregulates expression of eNOS and release of NO as a potent vasodilator (Loufrani & Henrion, 2008). In bovine aortic endothelial cells, 6h exposure to shear stress of 4 and 25 dynes/cm^2^ induced eNOS mRNA levels by 4.8‐ and 7.95‐fold, respectively, compared with stationary control (Xiao et al., 1997). Previous reports have suggested that eNOS expression and, therefore, NO production is rapidly upregulated in bovine aortic endothelial cells within 30 min of shear stress exposure (Bretón‐Romero et al., 2012). Further reports indicate that shear stress induces the phosphorylation of a tyrosine residue that negatively regulates eNOS activity, which is speculated to play a key role in limiting enzyme activity, thus keeping NO output low and reducing the risk of cofactor, tetrahydrobiopterin, depletion, and the uncoupling of the enzyme, while preventing NO‐mediated toxicities such as peroxynitrite formation (Fisslthaler et al., 2008). Together, these findings confirm that our study achieved a model of chronic shear stress in which enhanced NO generation stimulated by shear stress may reduce ET‐1 expression in an autoregulatory fashion (Ballermann et al., 1998).

Disruption of the actin cytoskeleton in CF epithelial cells has been documented (Castellani et al., 2017), which is restored in the presence of CFTR modulators (Abbattiscianni et al., 2016). However, only a single study has reported actin stress fiber formation following CFTR inhibition using both GlyH‐101 or CFTRinh‐172 in rat lung microvascular endothelial cells (Brown et al., 2014). The present data are the first in HLMVEC that demonstrates exacerbated F‐actin formation following GlyH‐101 treatment, which was characterized by a lack of cortical rim F‐actin and the formation of cytosolic stress fibers (Figure 4). TNF‐α treatment‐induced actin polymerization in both static and shear stress conditions, without significant redistribution of peripheral F‐actin (Figure 4). TNF‐α was previously shown to increase F‐actin and morphological changes in the endothelium through activation of GTPase RhoA‐mediated signalling to Rho kinase (ROCK), whereas TNF‐induced intracellular reactive oxygen species (ROS) were proposed to play a role in loss of endothelial barrier function through destabilization of cell‐cell contacts (Marcos‐Ramiro et al., 2014). However, we previously showed GlyH‐101 induces H_2_O_2_ release in HLMVEC cultures (Khalaf et al., 2020), and since H_2_O_2_ similarly causes actin filament rearrangement with disruption of the dense peripheral bands and formation of stress fibers in HUVECs (Huot et al., 1997) and in bovine pulmonary artery endothelial cells (Zhao & Davis, 1998), a role for H_2_O_2_ in the F‐actin responses to CFTR inhibition in HLMVECs may be indicated and requires further study.

Interestingly, significantly increased actin polymerization and the formation of paracellular gaps in response to flow was only observed in circumstances of CFTR inhibition (Figure 4g). Given that the actin cytoskeleton is a first‐responder to shear stress (Loufrani & Henrion, 2008) and facilitates the reformation of cells to align in the direction of flow (Osborn et al., 2006), a loss of CFTR activity could contribute to reduced endothelial barrier function observed in people with CF (Declercq et al., 2021) through altered actin dynamics and a loss of cellular alignment in response to flow.

Disturbed flow‐induced changes to endothelial cell morphology is an in vivo (Dolgov et al., 1982; Goode et al., 1977) and in vitro (Davies, 1995) marker of endothelial dysfunction. Shear stress increases the expression of VEGF and VEGF receptor 2 (VEGFR2; dela Paz et al., 2012), which promotes actin polymerization (Rousseau et al., 2000) and cell elongation through Rho/Rho‐associated protein kinase signalling during angiogenesis (Tsuji‐Tamura & Ogawa, 2018). Our laboratory has previously demonstrated that CFTR inhibition significantly increased VEGF expression in HLMVEC (Khalaf et al., 2020). However, the present study demonstrated that GlyH‐101 treatment significantly reduced HLMVEC alignment in the direction of flow and also promoted cellular retraction compared to both vehicle control and TNF‐α (Figure 2). Previous research has also observed that CFTRinh‐172 treatment caused HUVEC retraction and shrinkage, membrane bleb formation, and cellular vesicle release in response to shear stress (5 dynes/cm^2^; Totani et al., 2017). Reduced alignment in the direction of flow following CFTR blockade, despite elevated VEGF expression (Khalaf et al., 2020) and unchanged [nitrite]/[ET‐1], suggests that a factor downstream from VEGF is altered in this model of the CF endothelium.

Forkhead box protein O1 (Foxo1) is activated through VEGF‐induced/PI3K Akt‐signalling and modulates endothelial cell specification, growth, and actin remodeling (Tsuji‐Tamura & Ogawa, 2018). Preliminary reports have suggested that a loss of CFTR reduces Foxo1 expression in CF epithelial cells, independent of PI3K/Akt expression, which was rescued following treatment with IGF‐1 (Smerieri et al., 2014). However, no such data exists with regard to endothelial cell culture. Notably, in circumstances of increased VEGF, actin remodeling to form cytosolic stress fibers is prevented by the downregulation of mammalian target of rapamycin (mTOR) complex 2 (mTORC2) and its mTOR‐independent companion of complex 2 (Rictor; Farhan et al., 2015). CF epithelial cells have an increased expression of both mTOR and Rictor (Reilly et al., 2017), which could explain the disturbed morphology and loss of cortical rim F‐actin in epithelial cells (Abbattiscianni et al., 2016; Castellani et al., 2017) and possibly also in HLMVEC treated with GlyH‐101 observed in the present study; however, this warrants further investigation. Furthermore, both the upregulation of Rictor in HUVEC (Yang et al., 2018) and inhibition of CFTR in HLMVEC (Khalaf et al., 2020) is associated with suppressed Nrf2 expression and increased intracellular ROS content. Suppression of Nrf2 is likely to contribute to the systemic pro‐oxidant state people with clinically stable CF lung disease experience (Causer et al., 2020), which may also be associated with changes in transcriptional regulation of cytoskeleton components and disturbed cytoskeleton signalling (Cho et al., 2005; Dominguez & Holmes, 2011). Importantly, clinically approved CFTR modulator therapies restore Nrf2 transcription and activity in CF epithelial cells (Borcherding et al., 2019), and provide an exciting rationale to investigate potential effects of CFTR modulator therapy on the defective functions of the CF endothelium. In this context, Declerq et al. (2021) recently reported that the combination of CFTR modulators lumacaftor and ivacaftor (Orkambi®), when applied at concentrations of 3 µM, partially rescued the pro‐inflammatory phenotype of blood outgrowth endothelial cells derived from CF patients with severe disease. Further, lumacaftor restored the localization of F‐actin at the apical membrane of polarized CF bronchial epithelial cells (Abbattiscianni et al., 2016). Although the effect of CFTR modulators on the distribution of F‐actin and endothelial barrier function in primary CF endothelial cells remains to be investigated, evidence is accumulating that the endothelium is potentially a target for CFTR modulator therapy for patients with CFTR mutations for whom specific modulators are available. However, since more than 10% of CFTR mutations do not produce any CFTR protein for CFTR modulators to act upon, (Fajac & Sermet‐Gaudelus, 2021), other approaches are required (Yang et al., 2021).

There are limitations in our study to point out. In addition to the lack of F‐actin data at the 48h time point, the analysis of F‐actin expression was semi‐quantitative only and it would be useful to confirm the data with quantitative analysis. Further, the specificity of the selected CFTR inhibitors (GlyH‐101 and CFTRinh‐172) has been questioned (Kelly et al., 2010), due to reports that it may increase mitochondrial ROS production independent of changes in CFTR function. However, data from our laboratory have recently demonstrated that a mitochondrial‐specific antioxidant (MitoQ) had no effect on ROS generation in HLMVECs following GlyH‐101 treatment (Khalaf et al., 2020). Furthermore, following GlyH‐101 treatment CFTR is still expressed at the apical membrane but is not functional (i.e., a class III‐V mutation) (Verkman et al., 2013). However, a recent study has developed a long‐term cell culture of CF (p.Phe508del) HPAEC (Plebani et al., 2017); therefore, it would be useful to confirm the findings of the present study in cells that represent a loss of CFTR at the apical membrane (i.e., class I–II mutations; Veit et al., 2016).

To conclude, the present study has demonstrated that endothelial CFTR regulates actin cytoskeleton remodeling and promotes morphological alignment with flow in HLMVEC, independent of nitrite and ET‐1 pathways. Additionally, actin polymerization following endothelial CFTR inhibition was characterized by a loss of cortical rim F‐actin and the development of cytosolic stress fibers. Finally, HLMVEC exposed to flow following CFTR inhibition had a greater variability in cellular alignment, which could have implications for endothelial barrier permeability and function in vivo. Further experiments using CFTR knock‐out cells and CFTR siRNA are needed to support the ascribed endothelial CFTR functions.

## CONFLICT OF INTEREST

There are no conflict of interest to report.

## AUTHOR CONTRIBUTION

Janis K. Shute devised the study; Adam J. Causer, Maha Khalaf, Emily Klein Rot, Kimberly Brand, James Smith, and Janis K. Shute conducted the cell culture, staining, and imaging experiments and analysis of the images using ImageJ. Maha Khalaf analyzed ET‐1, and Stephen J. Bailey analyzed nitrite in the samples. All authors contributed to the drafting, writing, and review of the manuscript.

## Supporting information



Fig S1

Fig S2
