# Supplementary material for: CFTR limits F‐actin formation and promotes morphological alignment with flow in human lung microvascular endothelial cells
Source: Physiol Rep. 2021 Dec 1;9(23):e15128. doi: 10.14814/phy2.15128 (PMC8634629; doi:10.14814/phy2.15128)
Supplement: Supplementary file 1 — Fig S1 [file PHY2-9-e15128-s002.docx]

Figure S1.


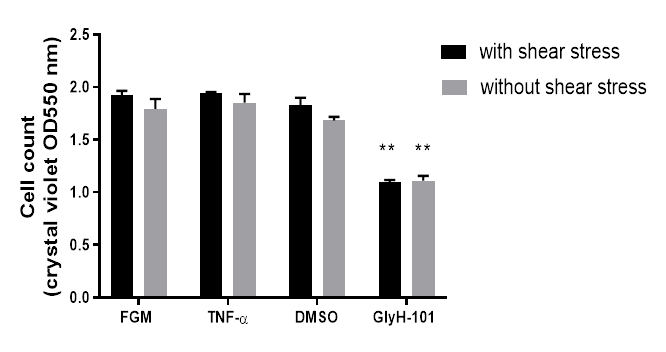


**HLMVEC cell viability after 48 h incubation**:

HLMVEC were treated for 48 h, with and without exposure to shear stress, with TNF-α (10 ng/ml) or GlyH-101 (20 µM) or with their appropriate controls (FGM or 0.1%DMSO respectively).

Cell viability was assessed indirectly as the number of adherent cells by staining with crystal violet (0.125% (w/v) in water) for 10 minutes, washing 3 times in phosphate buffered saline and extracting the stain with ethanol/acetone (80/20%) for 5 minutes. The OD 550 nm of this extract was compared under each condition.

The CFTR inhibitor significantly decreased the cell viability in both the absence and the presence of shear stress after 48 h (** p value ˂ 0.01 comparing GlyH-101 with DMSO, and with TNF-α, n=3). Data are expressed as mean ± SEM, and analysed with two-way ANOVA and Tukey’s multiple comparison test. FGM; full growth medium.
