# Supplementary material for: CFTR limits F‐actin formation and promotes morphological alignment with flow in human lung microvascular endothelial cells
Source: Physiol Rep. 2021 Dec 1;9(23):e15128. doi: 10.14814/phy2.15128 (PMC8634629; doi:10.14814/phy2.15128)
Supplement: Supplementary file 2 — Fig S2 [file PHY2-9-e15128-s001.docx]

Figure S2.

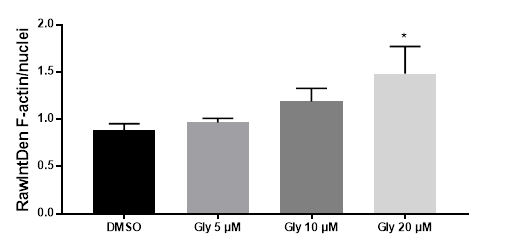
A. HLMVECs stained for F-actin following treatment with the CFTR inhibitor GlyH-101 in the concentration range 5, 10, 20 µM for 16h.

B. HLMVECS stained for F-actin and Hoechst blue were quantified after 16 h treatment with CFTR inhibitor GlyH-101 (5, 10 and 20 µM), (* p value ˂ 0.05, n=3). Data expressed as mean ± SEM. Data were analysed with one-way ANOVA and Fisher test.

**F-actin response to increasing concentrations of GlyH-101 over 16h:**

Cells stained with rhodamine phalloidin appeared to show a concentration-dependent increase in the intensity of F-actin stress fibre formation with GlyH-101 (Figure S2A). However, analysis of the data from 3 independent experiments showed that only the highest concentration of GlyH-101, 20 µM, increased the amount of F-actin in the cells (Figure S2B).

The raw integrated density (RawIntDen) of F-actin staining (Rhodamine Phalloidin) was measured, then normalised to the raw integrated density of nuclei (Hoechst stain).

For control HLMVECs treated with DMSO 0.1% value, the RawIntDen F-actin/nuclei was 0.89 ± 0.07. CFTR inhibitor GlyH-101 at 5 and 10 µM concentration did not significantly change RawIntDen F-actin/nuclei (0.97 ± 0.04 and 1.19 ± 0.14) respectively. However, GlyH-101 at 20 µM significantly increase RawIntDen F-actin/nuclei to 1.48 ± 0.29. Data represent 3 independent experiments.
